# Evaluation of MLC leaf positioning accuracy for static and dynamic IMRT treatments using DAVID *in vivo* dosimetric system*

**DOI:** 10.1120/jacmp.v17i2.5474

**Published:** 2016-03-08

**Authors:** Gulay Karagoz, Faruk Zorlu, Mete Yeginer, Demet Yildiz, Gokhan Ozyigit

**Affiliations:** ^1^ Department of Radiation Oncology Faculty of Medicine Hacettepe University Ankara Turkey; ^2^ Cancer Institute Hacettepe University Ankara Turkey

**Keywords:** leaf positioning, IMRT, DAVID, correlation analysis

## Abstract

Accuracy and precision of leaf positioning in multileaf collimators (MLCs) are significant factors for the accuracy of IMRT treatments. This study aimed to investigate the accuracy and repeatability of the MLC leaf positioning via the DAVID *in vivo* dosimetric system for dynamic and static MLC systems. The DAVID system was designed as multiwire transmission ionization chamber which is placed in accessory holder of linear accelerators. Each wire of DAVID system corresponds to a MLC leaf‐pair to verify the leaf positioning accuracy during IMRT treatment and QA. In this study, verifications of IMRT plans of five head and neck (H&N) and five prostate patients treated in a Varian DHX linear accelerator with 80‐leaf MLC were performed using DAVID system. Before DAVID‐based dosimetry, Electronics Portal Imaging Device (EPID) and PTW 2D ARRAY dosimetry system were used for 2D verification of each plan. The measurements taken by DAVID system in the first day of the treatments were used as reference for the following measurements taken over the next four weeks. The deviations in leaf positioning were evaluated by “Total Deviation (TD)” parameter calculated by DAVID software. The delivered IMRT plans were originally prepared using dynamic MLC method. The same plans were subsequently calculated based on static MLC method with three different intensity levels of five (IL5), 10 (IL10) and 20 (IL20) in order to compare the performances of MLC leaf positioning repeatability for dynamic and static IMRT plans. The leaf positioning accuracy is also evaluated by analyzing DynaLog files based on error histograms and root mean square (RMS) errors of leaf pairs’ positions. Moreover, a correlation analysis between simultaneously taken DAVID and EPID measurements and DynaLog file recordings was subsequently performed. In the analysis of DAVID outputs, the overall deviations of dynamic MLC‐based IMRT calculated from the deviations of the four weeks were found as 0.55%±0.57% and 1.48%±0.57% for prostate and H&N patients, respectively. The prostate IMRT plans based on static MLC method had the overall deviations of 1.23%±0.69%, 3.07%±1.07%, and 3.13%±1.29% for intensity levels of IL5, IL10, and IL20, respectively. Moreover, the overall deviations for H&N patients were found as 1.87%±0.86%, 3.11%±1.24%, and 2.78%±1.31% for the static MLC‐based IMRT plans with intensity levels of IL5, IL10 and IL20, respectively. Similar with the DAVID results, the error rates in DynaLog files showed upward movement comparing the dynamic IMRT with static IMRT with high intensity levels. In respect to positioning errors higher than 0.005 cm, static prostate IMRT plans with intensity levels of IL10 and IL20 had 1.5 and 2.6 times higher error ratios than dynamic prostate IMRT plans, respectively, while these values stepped up to 8.4 and 12.0 for H&N cases. On the other hand, according to the leaf pair readings, reconstructed dose values from DynaLog files had significant correlation (r=0.80) with DAVID and EPID readings while a stronger relationship (r=0.98) was found between the two dosimetric systems. The correlation coefficients for deviations from reference plan readings were found in the interval of −0.21–0.16 for all three systems. The dynamic MLC method showed higher performance in repeatability of leaf positioning than static MLC methods with higher intensity levels even though the deviations in the MLC leaf positioning were found to be under the acceptance threshold for all MLC methods. The high intensity levels increased the positioning deviations along with the delivery complexity of the static MLC‐based IMRT plans. Moreover, DAVID and EPID readings and DynaLog recordings showed mutually strong correlation, while no significant relationship was found between deviations from reference values.

PACS number(s): 87.56.J‐

## I. INTRODUCTION

New technologies and imaging techniques have improved the quality of radiotherapy treatments while the need for detailed verification and quality assurance of treatment plans and the output of treatment machines has increased simultaneously. 3D imaging of a patient provided information about the positioning of the tumor and critical organs in its proximity in 360° view angle. Thus, 3D conformal radiotherapy became possible, with the freedom to select treatment field angles. Multileaf collimator (MLC) systems took subsequently the role of beam shaping, instead of custom blocks that needed a heavier labor force for making and shaping them and applying them in a treatment room.^1^, ^2^

Intensity modulated radiation therapy (IMRT) technique uses MLCs for modifying the beam fluence in the same treatment field in order to improve the conformity of prescribed dose distribution around the tumor region.^3^, ^4^, ^5^, ^6^ The conformal treatment via IMRT makes the dose escalation so possible that the normal tissue complication probability is kept low while tumor control probability is lifted to higher levels. IMRT achieves the dose modulation by means of two MLC‐based dose delivery methods (i.e., dynamic MLC (dMLC) and step‐and‐shoot or static MLC (sMLC)). The dMLC method modifies the beam intensity by moving each MLC leaf with individual speed while the beam delivery continues. However, the sMLC method turns off the beam while the MLC leaves are moving. The treatment machine starts to shoot the beam only when the MLC leaves reach their calculated positions. The sMLC method forms discrete fluence maps with different numbers of intensity levels (ILs) to represent a targeted continuous fluence.^6^, ^7^

The MLC‐based beam delivery increases the complexity of a treatment and thus the need for patient‐specific quality assurance (QA) arises. Dosimetric measurements of a plan QA can be performed before and/or during the course of the treatment. Medical physicists routinely use various dosimetric systems to perform the pretreatment QAs such as the different types of ionization chambers, diodes, thermoluminescence dosimetry (TLD), 2D arrays, and films.^1^, ^2^, ^4^ However, these pretreatment measurements miss the possible variations that can emerge during the treatment course. *In vivo* dosimetry has, therefore, a unique place in the verification of dose delivered to the patient in a fraction of the therapy.

In this study, the Device for the Advanced Verification of IMRT Delivery (DAVID) (PTW, Freiburg, Germany) is employed to assure the quality of the delivered dose during the course of treatment.^(8)^ The rationale of the system is to check the position of each MLC leaf pair by measuring the delivered dose in each fraction and to compare it with the reference values (e.g., those acquired in the first day of the treatment). By the way, the system can follow the repeatability of the MLC‐based IMRT plans from the second day of the treatment to the last day.

## II. MATERIALS AND METHODS

### A. DAVID *in vivo* system and measurements

The verifications of leaf positioning of IMRT plans were performed by DAVID *in‐vivo* dosimetric system that was designed for online monitoring the delivered dose and comparing it with reference data. The device was placed into the accessory tray in the linac head. The DAVID system measured the delivered dose by a set of multiwire ion chambers in the same number of MLC leaf pairs embedded in the linac. Each wire of the system was assigned to a leaf pair to check its positioning accuracy. The readings in the device were transmitted to the DAVID software system via Bluetooth, in which the instantaneous measurement of each wire was compared with the reference value taken in the first day of the treatment. The software reported the misplacement of the MLC leaves as deviations.^8^, ^9^

The dosimetric measurements were performed using 6 MV photon beams generated by a Varian Clinac DHX linear accelerator (Varian Medical Systems, Palo Alto, CA) with 80‐leaf MLC system installed in the department of Radiation Oncology at Hacettepe University. The MLC positioning verification of five head and neck (H&N) and five prostate IMRT plans were performed using DAVID system. The planning target volumes (PTVs) and critical structures of IMRT plans were delineated by experienced radiation oncologists based on ICRU Report 83.^(10)^ The H&N plans had the organs at risk of spinal cord, brain stem, chiasm, parotid glands, optical nerves, eyes, esophagus, larynx, lip, oral cavity, mandibula and temporomandibular structures, while the critical structures (e.g., bladder, rectum, and femur heads) were contoured for the prostate plans. The treatment plans were prepared in Eclipse TPS (Varian) considering the dose tolerances of critical organs as reported by the RTOG (Radiation Therapy Oncology Group).^(11)^ As recommended in ICRU Report 62,^(12)^ the minimum and maximum doses of PTV were kept between the dose thresholds of 95% and 107% of prescribed dose, respectively.

The delivered IMRT plans were originally prepared using the dMLC method and then the same plans were calculated using the sMLC method in order to compare the MLC positioning performances of dynamic and static IMRT plans. The static plans were prepared for three intensity levels (ILs) of 5, 10, and 15. The measured dose for each pair of the MLC was recorded as the reference value for the four next weeks in order to compare and verify the leaf positioning over the course of the treatment. The percentile deviation (”Total Deviation” (TD)) of each week was reported for each dMLC and three sMLC plans of five prostate and five H&N patients.

### B. Pretreatment plan verification

The 2D dose verifications of the IMRT plans were performed using both the electronic portal imaging device (EPID) embedded in the treatment machine and 2D ARRAY seven29 dosimetry system (PTW, Freiburg, Germany). The calculated dose maps in TPS systems were compared with the dose maps measured by these two dosimetry systems using gamma analysis with the acceptance criteria of 3% dose difference and 3 mm distance to agreement.^(13)^ Moreover, the MLC positioning accuracy of the linac was also checked using dynamic MLC log (DynaLog) files that are generated for Millennium MLC sets (Varian). DynaLog files compared the actual positions of leaf pairs during delivery with the targeted positions in the treatment plan. The sampling frequency of the DynaLog files in MLC position recording was $20\;s^{-1}$. The error rates were analyzed using DynaLog file viewer software.

### C. Correlation analysis

The correlations between DAVID and EPID readings and DynaLog recordings were also analyzed in order to investigate the coherence between dose reports of the systems. For this purpose, the EPID system was located at source‐to‐imager distance (SID) of 105 cm while the DAVID system was placed into the accessory tray. The two dynamic plans, one H&N and one prostate, were delivered more than 15 times while the two dosimetric systems took the dose measurements simultaneously. The dose readings and recordings corresponding to each of the leaf pairs were extracted from the acquired data of DAVID, EPID and DynaLog files. The data analyses were performed in a MATLAB (MathWorks, Natick, MA) programming language environment. EPID data matrix for each treatment field consisted of 512×384 dose readings of the aSi detectors. Since the projection width of each leaf pair covered over almost 13 detectors at the SID of 105 cm, the cumulative dose of a leaf pair was calculated by averaging the dose readings of corresponding detector set. DynaLog files contained the information of actual positions of leaf banks A/B and dose fractions. The leaf gaps and corresponding dose rates were calculated using the data and thus the cumulative dose of each leaf pair was estimated. After elimination of the dose readings lower than 5% of the maximum dose, a total number of 1,995 leaf pair readings were analyzed to find the correlation between the datasets. The deviation from reference value was also calculated for each leaf pair reading and correlation analysis was performed. The absolute value of correlation coefficient higher than 0.5 was accepted as strong at the end of the analysis.

## III. RESULTS

**Figure 1:**
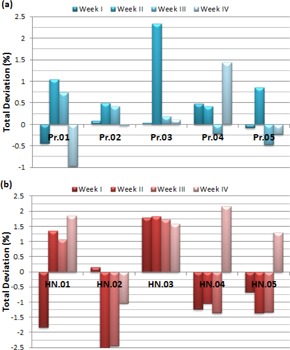
The weekly deviations of five prostate (a) and five H&N (b) patients for dynamic MLC‐based IMRT plans. Pr=Prostate patient; HN=Head&Neck patient.

The MLC leaf repetition performance of dynamic IMRT treatment is summarized in Fig. 1, in which deviations between the reference readings of the first day and the testing readings of the next four weeks are reported for five prostate and five H&N patients. The absolute mean of percentile deviations for the prostate plans are 0.22%±0.22%, 1.03%±0.77%, 0.41%±0.22%, and 0.56%±0.62% for from 1st week to 4th week, respectively. The H&N plans have higher absolute means for the four weeks from the first to the last as 1.13%±0.72%, 1.62%±0.57%, 1.58%±0.53%, and 1.58%±0.44%, respectively. The overall absolute means of deviations of the dynamic plans are 0.55% and 1.48% for the prostate and H&N patients, respectively.

**Figure 2:**
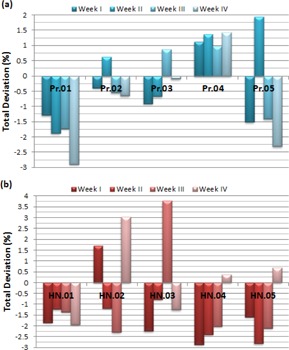
The weekly deviations of five prostate (a) and five H&N (b) patients for static MLC‐based IMRT plans with five intensity levels. Pr=Prostate patient; HN=Head&Neck patient.

Figure 2 shows the deviations in the verification of sMLC plans with five intensity levels (IL5). The deviations for prostate plans are in the band of −3% and 2% whereas the H&N plans have the deviations in the band of −3% and 4%. The absolute means of sMLC_IL5 plans for the prostate patients are 1.04%±0.43%, 1.29%±0.63%, 1.11%±0.47% and 1.48%±1.15% for from Week I to Week IV, respectively. Similar to the dMLC plans, three‐quarters of sMLC_IL5 plans for the H&N patients have higher average deviations as 2.05%±0.52%, 1.68%±0.88%, 2.31%±0.89%, and 1.46%±1.06% for the first to last week, respectively. The overall average deviation of prostate plans is 33% lower than the H&N deviations.

**Figure 3:**
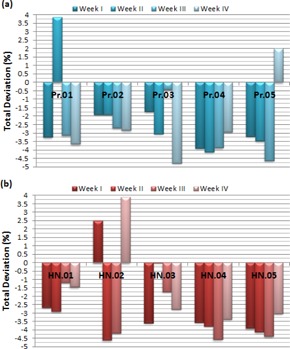
The weekly deviations of five prostate (a) and five H&N (b) patients for static MLC‐based IMRT plans with 10 intensity levels. Pr=Prostate patient; HN=Head&Neck patient.

The results of repeatability analysis of sMLC_IL10‐based IMRT plans are depicted in Fig. 3. The intervals of the deviations are almost the same for the prostate and H&N plans. The average deviations of prostate plans are in the interval of 2.79%‐3.28% while the interval of average H&N deviations is 2.90%‐3.24%. The overall averages are 3.07%±1.07% and 3.11%±1.24% for prostate and H&N plans, respectively. The sMLC_IL10‐based plans show higher deviations than both dMLC and sMLC_IL10 plans.

**Figure 4:**
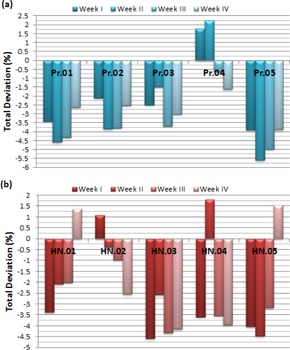
The weekly deviations of five prostate (a) and five H&N (b) patients for static MLC‐based IMRT plans with 20 intensity levels. Pr=Prostate patient; HN=Head&Neck patient.

Figure 4 represents the deviations of IMRT plans based on sMLC technique with 20 intensity levels (sMLC_IL20). The weekly average deviations for the prostate patients are 2.74%±0.89%, 3.55%±1.68%, 3.48%±1.68%, and 2.74%±0.82% for the weeks of I, II, III, and IV, respectively. The H&N plans show the average deviations of 3.34%±1.34%, 2.26%±1.48%, 2.80%±1.31%, and 2.71%±1.31% for the weeks in the same order, respectively. The overall averages of deviations are 3.13%±1.29% and 2.78%±1.31% for the prostate and H&N patients, respectively.

Comparing the dMLC‐ and sMLC_IL5‐based IMRT plans, the sMLC‐based plans with higher intensity levels show higher average deviations for both the prostate and H&N patients. The average deviations of the sMLC_IL10 and sMLC_IL20 plans are more than fivefold greater than the deviations of the dMLC‐based prostate plans. This ratio reduces to almost twofold for H&N patients. The average deviations of sMLC_IL5‐based prostate and H&N plans are almost 40% and 60% of deviations of sMLC‐based plans with higher intensity levels, respectively.

The analyses of leaf positioning error based on the DynaLog files of the dynamic and static IMRT plans are summarized in Table 1. The error value of 0.005 cm was accepted as the upper threshold for counting the erroneous positioning as correct. Similar to the DAVID measurements, the ratio of errors higher than the threshold of 0.005 cm shows upward movement with the increase in the intensity level of the static IMRT plans from IL5 to higher levels. DynaLog files report 6.0 and 1.8 times more error rates (>0.005 cm) for sMLC_IL20‐based prostate plans than the plans with five and ten intensity levels, respectively. H&N plans with five intensity levels similarly have lower reported error ratios (>0.005 cm) than treatment plans with higher intensity levels. Comparing with dMLC plans, static IMRT plans with 10 and 20 intensity levels have 1.5 and 2.6 times higher ratios of error (>0.005 cm) for prostate cases, respectively. These values reach to 8.4 and 12.0 for H&N cases. On the other hand, average RMS errors of dMLC plans stay in the lower level of 0.02‐0.11 cm while the static plans have values in the range of 0.14‐0.18 cm. Maximum RMS errors of the leaf pairs are in the ranges of 0.03‐0.30 cm and 0.03‐0.04 cm for dynamic prostate and H&N plans, respectively. These ranges extend to 0.19‐0.40 cm and 0.19‐0.39 cm for sMLC_IL20‐based prostate and H&N plans, respectively.

The results of Gamma analyses between the calculated 2D dose distributions in Eclipse TPS system and the measured dose maps taken by EPID and PTW 2D ARRAY dosimetry systems are listed in Table 2. These values are separately averaged for the dynamic and static IMRT plans of the patients. The error rates of EPID‐based QA measurements are lower than 3% for the four IMRT plans of the all prostate and H&N patients. As expected, the error rates of 2D ARRAY measurements are generally higher than the rates of the EPID, while they stay in the error threshold of 5%. The upward trends in the error rates are also observed in 2D ARRAY measurements from dynamic to static plans with high intensity levels.

**Figure 5:**
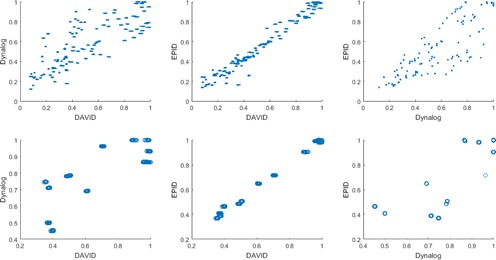
The distributions of 1,995 leaf pair readings (1st row) and 237 field total doses (2nd row) for the three systems of DAVID, EPID, and DynaLog files.

The distributions of normalized leaf pair readings are plotted in the first row of Fig. 5. The dose readings of DAVID and EPID systems and the estimated dose values acquired from DynaLog files are analyzed to investigate the correlation between the dose sets. A total number of 1,995 leaf pair readings from 237 treatment fields for each dosimetric system construct the data matrices after elimination of dose readings lower than 5% of maximum dose values. The Pearson analysis shows strong correlations between the three dose sets. The DynaLog set had the same level of correlation (r=0.80) as the DAVID and EPID sets, while the DAVID set showed stronger relationship (r=0.98) with the EPID set. Analyzing of total dose values of 237 treatment fields gives similar correlations between the dosimetric systems. The second row of Fig. 5 demonstrates the relationship between the total dose values of the three sets. The DAVID and EPID systems show again strong correlation with Dynalog files with *r* values of 0.78 and 0.74, respectively, while the correlation between DAVID and EPID total doses is higher (r=0.997). Correlation of deviations from the reference plans are also analyzed using the leaf pair readings of the three systems. The deviation sets show no correlation for any couple of the three systems such that the correlation coefficients are in the range of ‐0.21 and 0.16.

**Table 1:** Average error ratios less than 0.005 cm and average RMSE values calculated based on DynaLog files of dynamic and static IMRT plans of the five prostate and five Head&Neck patients

		*dMLC*	*sMLC_IL5*	*sMLC_IL10*	*sMLC_IL20*
Error<0.005 cm (%)	Prostate	98.5±0.6	99.3±0.5	97.7±0.5	95.9±1.1
	H&N	99.7±0.2	98.9±0.5	97.4±0.4	96.4±0.8
Average RMSE (cm)	Prostate	0.11±0.10	0.14±0.07	0.15±0.02	0.18±0.08
	H&N	0.02±0.00	0.17±0.03	0.15±0.04	0.18±0.06

dMLC=dynamic multileaf collimator technique; sMLC_IL#=static MLC technique with # intensity levels; RMSE=root mean square error.

**Table 2:** Gamma analysis results of EPID and 2D ARRAY dosimetry systems for dynamic and static IMRT plans of the five prostate and five Head&Neck patients

		*dMLC*	*sMLC_IL5*	*sMLC_IL10*	*sMLC_IL20*
EPID	Prostate	99.0±0.4	98.3±0.4	98.8±0.4	98.3±0.7
(%)	H&N	99.0±0.7	98.6±0.8	98.8±0.7	98.0±0.6
2D ARRAY	Prostate	98.6±1.5	98.2±1.9	96.2±1.7	94.5±1.9
(%)	H&N	96.4±1.9	96.3±1.8	94.3±2.1	91.8±1.7

dMLC=dynamic multileaf collimator technique; sMLC_IL#=static MLC technique with # intensity levels.

## IV. DISCUSSION

The complexity of IMRT treatments requires special attention in each step of planning, dosimetry, patient setup and dose delivery. Patient‐specific pretreatment IMRT QA is a routine in radiotherapy facilities now. Even if the dose constraints in the treatment planning system and the pretreatment dosimetric measurements approve the treatment plan, interfractional variations over the course of the treatment can only be followed with *in vivo* dosimetry. Diverse types of dosimetric systems (e.g., TLDs, diodes, films, ionization chambers, and EPID) can be employed for *in vivo* dosimetry.^(2)^

In this study, DAVID, a transmission‐type multiwire ion chamber system was used to check the dose fluence from each channel of MLC leaf pairs and thus to evaluate the leaf positioning performances of IMRT treatments. Before the IMRT verification measurements, the permeability of the DAVID system to the transmitted photon beam was also evaluated. The transmission factor was found by comparing the doses with and without DAVID system. The measurements were taken for four square fields at a source‐to‐surface distance (SSD) of 100 cm and a depth of 1.5 cm ($d_{\text{max}}$). The transmission factors were calculated for the square fields of 5×5, 10×10, 20×20, and $40\times 40\;{\text{cm}}^{2}$ as 0.925, 0.927, 0.934, and 0.948, respectively. The measurements were repeated for the same four fields at the SSD of 90 cm and at the depth of 10 cm in order to evaluate the effect of the depth on the transmission factor. The factors were calculated as 0.929, 0.930, 0.934, and 0.944 for the same order of the four fields. The transmission factor of the system was taken as 0.935 in our measurements. Poppe et al.^(8)^ measured the transmission factors for 3D conformal radiotherapy technique in a Siemens linac. They found the factor as 0.953 and 0.968 for 6 MV and 18 MV nominal photon energies, respectively. The same research group performed another study on DAVID system in a Siemens linac and calculated the transmission factor as 0.934 for 6 MV photon beams.^(9)^ These results show that the transmission factor should be calculated for each energy option of the treatment machine.

Another important issue before our IMRT verification measurements was the error‐detection ability of the system. For this purpose, the readings of 20×20 cm open field were recorded as reference values in DAVID system. For testing setup, positions of two MLC pairs were changed as 1 mm and one pair was also slid as 3 mm. The readings of each test setup were then compared with the reference readings. The maximum deviation was calculated as −5.10% for 3 mm slid MLC pair, while deviations of −1.40% and −1.19% were found for 1 mm slid pairs. A negative sign represents that the dose measured in the test did not reach the reference value.

The homogeneity indices (HIs) of PTV dose distributions were also investigated for the delivered dMLC and sMLC‐based IMRT plans. This quality factor was calculated based on the definition of RTOG in which HI was defined as the ratio of the maximum PTV dose to the prescribed dose.^(11)^ The average HIs of the prostate patients were calculated as 1.051±0.016, 1.120±0.026, 1.060±0.013, and 1.051±0.014 for the methods of dMLC, sMLC_IL5, sMLC_IL10, and sMLC_IL20, respectively. Moreover, The H&N patient group had the average HIs of 1.056±0.010, 1.112±0.024, 1.057±0.014, and 1.051±0.011 for the same order of the methods. For the both patient groups, sMLC_IL5 showed higher heterogeneity in PTV dose distribution than the others. These results are supported by the study performed by Shwetha et al.^(14)^ in which the performances of dynamic and static MLC‐based IMRT and high‐dose‐rate brachytherapy were compared for cervical carcinoma. They found that the IMRT plans based on sMLC_IL5 had the lowest HI performance with the average HI of 1.29±0.13 while the other IMRT plans based on dMLC, sMLC_IL10 and sMLC_IL20 had the average HI in the band of 1.19‐1.21.

The correlation analysis between DAVID readings and the outputs of other dosimetric systems was, as far as we know, first performed in our study. There are, moreover, several studies in the literature that compared other dosimetric systems and analyzed the correlation between their readings. Agnew et al.,^(15)^ for instance, proposed a DynaLog‐based QA procedure for volumetric‐modulated arc therapy in order to reduce the QA workload for physicists. For this purpose, they analyzed the correlation between the gamma analysis results of pretreatment ionization chamber array and on‐treatment trajectory log files. They found a significant correlation (r=0.623) between them. Their correlation result was coherent with our correlation values between the total dose estimations from DynaLog files and the dose readings of DAVID (r=0.78) and EPID (r=0.74) systems. Calvo‐Ortego et al.^(16)^ also studied on DynaLog files to offer a new method for patient‐specific IMRT QA. They delivered several intended erroneous plans to compare the DynaLog‐based reconstructed fluences with EPID readings. The accuracy of reconstructed fluences was reported as comparable with EPIDs.

The sensitivity of dosimetric systems to MLC positioning error was another issue investigated in the literature. Vieillevigne at al.^(17)^ evaluated the performances of three QA systems (i.e., ArcCHECK, 2D Array 729, and EPID) in the detection of opening and closing MLC errors by gamma index comparison. They found that the three systems could detect 2 mm error with 3%/3 mm criteria while the detectable error value was reduced to 1 mm with 2%/2 mm. However, referring to DynaLog files in our study, 96.4% of the MLC positioning errors were lower than 0.5 mm, while the error ratios were 3.5% and 0.1% for the intervals of 0.5‐1.0 mm and 1.0‐1.5 mm, respectively. With the low level of positioning errors, the relationships between deviations from reference deliveries recorded by DAVID, EPID, and log files were not found correlated. The lack of evidence for correlation between the deviations could have arisen because the cumulative dose errors of leaf pairs may remain under an error level sufficient for revealing any relationship and performing sensitivity comparison. The sensitivity analysis of DAVID and other 2D dosimetric systems can be investigated in a further study in which treatment plans with gradually increased intended errors are delivered many times and dosimetrically analyzed.

## V. CONCLUSIONS

Comparing the sMLC‐based IMRT plans with higher intensity levels, the deviations of leaf positioning between the reference values of the first day and the readings taken in the next four weeks were in a lower level for the dMLC and sMLC_IL5 methods. The tumor localization, from prostate to H&N, showed its effect on the repeatability of the treatment more clearly in dMLC and sMLC_IL5‐based IMRT plans. Furthermore, although the correlation analysis stated strong relationships between the readings of DAVID and EPID systems and DynaLog records, the deviations showed no significant correlation.

## ACKNOWLEDGMENTS

We thank Mike Hughes for sharing on the web his MATLAB codes for analyzing Varian DynaLog files. His codes made our work easier in dose estimation from DynaLog data.

## COPYRIGHT

This work is licensed under a Creative Commons Attribution 4.0 International License.
